# Differences among factors associated with tobacco product use among Black and White adolescents: A cross-sectional analysis of wave one of the PATH study 2013–2014

**DOI:** 10.18332/tid/161932

**Published:** 2023-05-05

**Authors:** Adriana Espinosa, Fiona N. Conway, Lesia M. Ruglass, Christine E. Sheffer

**Affiliations:** 1 Department of Psychology, The City College of New York, New York, United States; 2 Steve Hicks School of Social Work, University of Texas at Austin, Austin, United States; 3 Department of Health Behavior, Roswell Park Comprehensive Cancer Center, Buffalo, United States

**Keywords:** tobacco use, adolescent African Americans, public health, health disparity, minorities

## Abstract

**INTRODUCTION:**

Tobacco use remains a primary cause of health disparities between Black and White Americans. Current approaches have not improved tobacco-related racial health disparities. This study aimed to identify differences in factors associated with tobacco product use among Black and White adolescents.

**METHODS:**

This cross-sectional design used data from Wave One (2013–2014) of the Population Assessment of Tobacco and Health Study. Adolescents aged 12–17 years who identified as non-Hispanic Black or African American (n=1800) or non-Hispanic White (n=6495) were included. Primary outcomes were the ever use and current use of any tobacco products. Sociocultural, household environment, psychological, and behavioral factors were included. Logistic regressions, stratified by race, were used to determine significance. Dominance analysis was used to rank significant factors by their level of importance.

**RESULTS:**

Although there were many Black–White commonalities, there were also important differences. Black adolescents in the Northeast were more likely to have ever used tobacco compared to those in the South (OR=0.6; 95% CI: 0.6–0.7, p<0.001) and Midwest (OR=0.6; 95% CI: 0.5–0.7, p<0.001). White adolescents in the Northeast were less likely to use tobacco products than in other regions. Peer influences (OR=1.9; 95% CI: 1.1–3.2, p<0.05) were uniquely associated with ever use among Black adolescents. Access to tobacco in the home (OR=2.0; 95% CI: 1.4–3.0, p<0.001) and thinking that tobacco use would help reduce stress (OR=1.3; 95% CI: 1.1–1.6, p<0.01) were uniquely associated with current use among Black adolescents.

**CONCLUSIONS:**

There are significant Black–White differences in the factors associated with tobacco use. Factors uniquely associated with Black adolescent tobacco use should be considered in developing strategies to prevent adolescent tobacco use in Black communities.

## INTRODUCTION

Despite the remarkable success of US tobacco control efforts, tobacco use remains a primary cause of health disparities between non-Hispanic Black or African Americans (henceforth referred to as Black) and non-Hispanic White Americans (henceforth referred to as White)^1,2^. Although the overall prevalence of tobacco use among Black and White Americans remains similar, a significantly higher proportion of Black men (20.9%) smoke cigarettes than the general public^3^; and Black Americans overall experience a disproportionate burden of tobacco-related death and disease^4-7^. Chronic tobacco use, with its subsequent health consequences, is most often initiated during adolescence, making adolescence a critical time for preventing tobacco use^8^. However, tobacco use among adolescents has immediate health consequences as well^8^. In addition, exposure to nicotine, the addictive component in tobacco, during adolescence can result in neural adaptations that increase the risk for other substance use^9,10^. Thus, prevention of tobacco use among Black adolescents is vital for the health of Black communities.

Historically, a one-size-fits-all approach to public health and tobacco control has not improved tobacco-related disparities and racial health gaps in the US^11-13^. For example, Black Americans do not benefit equally from smoke-free policies, a well-established strategy to promote smoking cessation^14-16^. Similarly, higher parental socioeconomic status (SES; i.e. education and/or income), a well-established protective factor against White adolescent tobacco use, does not equally protect Black adolescents from tobacco use^17-20^. Moreover, recent findings indicate that Black adolescents have not shown the same overall decline in adolescent tobacco smoking from 1999 to 2018^21^. Beginning in the period 2011–2014, a trend emerged whereby older Black adolescents began to show an increase in tobacco use prevalence rates while White adolescents continued to show a downward trend. Evidence suggests that race-specific and/or sociocultural factors rather than socioeconomic factors or policies are responsible for these differences, but exactly what those factors are remains unclear^21^. While systematic reviews have shown that multicomponent tobacco use prevention programs delivered in different settings (e.g. school, family) are effective, the specific factors targeted in these interventions are unclear, and Black–White outcomes remain virtually unexplored^8,22^.

Black and White adolescents have different sociocultural and other experiences, which, logically, can lead to different responses to prevention strategies. This, in turn, can be difficult to remediate when the influence of sociocultural and other factors is unaccounted for or unknown^23^. Understanding variations and differences in the factors associated with tobacco product use among Black and White adolescents is an important step in the identification of intervention targets that might be particularly effective among Black adolescents and a first step in the development of culturally tailored prevention strategies. Culturally tailored tobacco use prevention strategies might be especially important for Black adolescents because current approaches do not appear to benefit Black adolescents equally^21^.

Because this study was focused on racial differences or disparities, we used the National Institute on Minority Health and Health Disparities Research Framework as a guide for the selection of factors. This Framework provides an evolving conceptualization of factors relevant to understanding health disparities and has been adapted in multiple studies to examine different populations, health behaviors, and conditions^24,25^. The domains include sociocultural factors (demographics and family, peer, and community norms), environmental or household factors (household policies, environment, and parental interactions), and behavioral factors (coping strategies, use of other substances, social network discussions). We adapted the Framework to accommodate psychological factors which have been shown to have an effect of tobacco use among adolescents^26^.

This study aimed to identify differences in the factors associated with tobacco product use among Black and White adolescents. We focus specifically on Black–White differences for several reasons. The historical context, experiences, and health consequences of tobacco use among Black Americans are distinct from those of other racial and ethnic groups. For instance, Black Americans have been uniquely impacted by racism and discrimination and uniquely targeted by the tobacco industry^27-30^. Tobacco use also uniquely contributes to a persistent Black–White gap in mortality^29,31,32^. White adolescents were used as the comparison group because historically, White Americans have comprised the statistical majority and the dominant culture in the US and have served as the ‘standard’ by which all other groups have been compared.

## METHODS

### Study setting, participants, and design

We used data from wave one of the Population Assessment of Tobacco and Health (PATH) Study collected from 12 September 2013 to 14 December 2014 to examine our aim in a cross-sectional study design. The PATH Study is a collaboration between the US Food and Drug Administration Center for Tobacco Products and the National Institutes of Health. The PATH Study is uniquely well-suited to examine differences in factors associated with tobacco product use among Black and White adolescents. The PATH Study is the largest multi-year nationally representative longitudinal cohort study of tobacco use behavior, attitudes and beliefs, and tobacco-related health outcomes among US youth and adults, and oversamples Black adolescents. PATH Study recruitment includes a stratified address-based, area-probability sampling design. Data were collected using computer–assisted self-interviews. Data from adolescents were supplemented with parent/guardian reports^33,34^. Only participants who identified as non-Hispanic were included to eliminate the potential confounding influence of ethnicity. Of the 8295 participants included in the study, 1800 (21.7%) identified as Black and 6495 (78.3%) identified as White. As indicated prior, the National Institute on Minority and Health Disparities Research Framework was used to guide the selection and organization of the items from the PATH Study data^24,25^.

### Primary outcomes

Ever use of each tobacco product was assessed by asking: ‘Have you ever tried/used [tobacco product], even one or two times?’. Participants who reported ever using a tobacco product were asked: ‘When was the last time you used [tobacco product], even one or two times?’. Current tobacco use was defined as the use of any tobacco product in the past 30 days.

### Sociocultural factors

Age and sex were categorized as 12–14 or 15–17, and male or female. Parental education included five categories ranging from ‘less than high school’ to ‘advanced degree’. US Census regions (Northeast, Midwest, South, West) were used to identify geographical regions.

Peer influences were assessed by asking: ‘If your best friend were to offer you [tobacco product], would you use it?’. Responses were categorized as: ‘probably or definitely yes’ or ‘probably or definitely no’. Perceived normative access to tobacco products was assessed by asking: ‘How easy do you think it is for people your age to buy tobacco products in a store?’. Response options were: ‘very easy’, ‘easy’, ‘difficult’ and ‘very difficult’, scored from 1 to 4, respectively.

### Household environment factors

Tobacco use in the home was assessed by asking parents/guardians whether or not ‘anyone who lives with you now does any of the following: smoke cigarettes, use smokeless tobacco, smoke cigars, cigarillos or filtered cigars, or use any other form of tobacco?’. Access to tobacco products was assessed by asking parents/guardians whether or not they ‘think cigarettes or tobacco might be available to [participant] at your home?’ or ‘ … at another parent’s home’. Home tobacco use policies were assessed by asking: ‘For tobacco products that are burned, such as cigarettes, cigars, pipes or hookah, which statement best describes the rules about smoking a tobacco product inside your home?’ and ‘Now think about other tobacco products that are not burned, like smokeless tobacco, dissolvable tobacco, and electronic cigarettes. Which statement best describes the rules about using these products inside your home?’. Responses were categorized as products ‘not allowed anywhere or at any time inside my home’ or ‘allowed in some places or at some time inside my home’ or ‘allowed anywhere and at any time inside my home’. Parental guidance about tobacco use was assessed by asking whether or not: ‘In the past 12 months, have your parents or guardians talked with you, even once, about not using any type of tobacco product?’.

### Psychological factors

Beliefs about the positive emotional consequences of tobacco use were assessed with four statements: ‘I think I would enjoy using tobacco’; ‘I think using tobacco would help me reduce or handle stress’; ‘I think using tobacco would help me calm me down when I am angry’; and ‘I think using tobacco would help me to feel more comfortable at parties’. Response options included ‘strongly agree’, ‘agree’, ‘disagree’ and ‘strongly disagree’, scored from 1 to 4, respectively. The presence of attention deficit hyperactivity disorder was assessed by asking parents whether or not the participant has ‘ever been told by a doctor or other health professional that [he/she] has ADHD or ADD’ (the acronyms stand for attention-deficit hyperactivity disorder and attention deficit disorder, respectively).

### Behavioral factors

Whether or not participants had engaged in physical aggression was assessed by asking: ‘When was the last time that you started physical fights with other people?’. If the participant had ever started fights, the response was categorized as ‘yes’. Whether or not participants had used alcohol or other substances was assessed by asking: ‘Have you ever used alcohol at all, including sips of someone’s drink or your own drink?’ and ‘Have you ever used the following: marijuana, hash, THC, or grass; cocaine or crack; stimulants like methamphetamine or speed; any other drugs like heroin, inhalants, solvents or hallucinogens, prescription drugs not prescribed to you such as Ritalin, Adderall, painkillers, sedatives, or tranquilizers?’. Whether or not participants had engaged in social network discussions involving tobacco use was assessed by asking: ‘Has anyone discussed tobacco products on your Facebook, Google Plus, MySpace, Twitter, or other social networking account?’.

### Statistical analysis

Participants were characterized using weighted frequencies, percentages, means and standard deviations. Significant differences between Black and White participants were examined with chi-squared tests of independence and independent samples t-tests. Bonferroni-corrected z-tests were used to locate significant differences among categorical factors with >2 levels. Analyses were carried out in STATA v.15.1 using recommended sampling weights in the estimation of variances and standard errors^33^.

Logistic regressions, stratified by race, examined associations between factors and primary outcomes. A variance inflation factor <2 was used to examine whether or not multicollinearity was a significant source of bias. Robust standard errors (SE) addressed slight deviations from model assumptions for statistical inferences^35^. SEs were clustered by region to control for within-region homogeneity.

Dominance analysis (DA) examined the relative influence of significant factors in the logistic regression models^36-39^. Using a multi-step iterative procedure, DA estimates subsets of regressions for every possible combination of factors from the original logistic regression model. Thus, a model with *k* predictors results in a total of 2^k^ - 1 regressions^36,38,40^. DA calculates a series of pairwise comparisons and ranks each predictor variable according to its relative importance for improving the model fit based on goodness-of-fit statistics^36,38,40^.

## RESULTS

Nearly one quarter (22.5%) of participants had ever used tobacco, and one in ten (9.7%) was currently using tobacco.

### Sociocultural factors

The sample included slightly more males than females but was relatively balanced in age. Over two-thirds of the parents/guardians (70.6%) had some college or a Bachelor’s or an advanced degree. The South (39.7%) and Midwest (26.2%) were more highly represented than the Northeast (18.1%) and West (16.0%). Less than 10% reported that they would use tobacco if asked by their best friend. About two-thirds of participants reported that it was ‘very difficult’ or ‘somewhat difficult’ to purchase tobacco.

### Household environment factors

Nearly 40% of participants lived with someone who uses tobacco. About one-quarter (22.8%) of parents reported that participants had access to tobacco in their parents’ homes. About 85% of participants reported that tobacco use of any kind was not allowed inside their homes. About half (49.7%) of participants reported that their parents discussed not using tobacco products with them in the past year.

### Psychological factors

Over 90% ‘disagreed’ or ‘strongly disagreed’ that they would enjoy tobacco use or that tobacco use would help them feel more comfortable at parties. About 85% ‘disagreed’ or ‘strongly disagreed’ that tobacco use would help reduce stress or help calm anger. About 16% of participants had been diagnosed with ADHD.

### Behavioral factors

About 40% of participants had used alcohol, 20% had used other substances, and 21% had ever initiated physical fights with others. Nearly one-quarter (24.6%) had discussed tobacco use on social media. Table 1 details the descriptive analysis of the factors and the differences between Black and White participants.

**Table 1:** Differences between non-Hispanic White and non-Hispanic Black adolescents from Wave One of the Population Assessment of Tobacco and Health Study collected 2013–2014

*Factors*	*All (N=8295) n (%)*	*Non-Hispanic White (N=6494) n (%)*	*Non-Hispanic Black (N=1801) n (%)*	*χ^2^/ t*	*V/d*
***Sociocultural factors***					
**Age** (years)					
12–14	4103 (49.5)	3196 (49.2)	908 (50.4)	0.06	0.003
15–17	4192 (50.5)	3297 (50.8)	893 (49.6)		
**Sex**					
Male	4279 (51.7)	3357 (51.8)	919 (51.3)	0.01	0
Female	3997 (48.3)	3123 (48.2)	875 (48.8)		
**Parental education level**					
<High school	964 (11.7)	625 (9.7)^a^	353 (19.8)^a^	258.79***	0.18
High school	1463 (17.7)	1106 (17.1)^b^	362 (20.3)^b^		
Some college	2855 (34.6)	2184 (33.8)^c^	677 (37.9)^c^		
Bachelor’s degree	1919 (23.3)	1655 (25.6)^d^	248 (13.9)^d^		
Advanced degree	1050 (12.7)	896 (13.9)^e^	146 (8.2)^e^		
**Geographical region**					
Northeast	1500 (18.1)	1221 (18.8)^f^	273 (15.2)^f^	500.03***	0.25
Midwest	2176 (26.2)	1824 (28.1)^g^	339 (18.8)^g^		
South	3291 (39.7)	2279 (35.1)^h^	1044 (58.0)^h^		
West	1327 (16.0)	1169 (18.0)^i^	145 (8.1)^i^		
**Would use tobacco if asked by best friend** (Ref. No)	762 (9.2)	593 (9.1)	169 (9.4)	0.03	0.002
**Difficulty purchasing tobacco**, mean score ± SD	2.83 ± 0.99	2.85 ± 0.98	2.77 ± 1.07	2.43*	0.06
**Difficulty purchasing tobacco** (score)					
Very easy (1)	910 (11.2)	659 (10.3)	257 (14.5)		
Somewhat easy (2)	2134 (26.2)	1653 (26.0)	482 (27.2)		
Somewhat difficult (3)	2508 (30.8)	2060 (32.4)	437 (25.6)		
Very difficult (4)	2588 (31.8)	1996 (31.3)	595 (33.6)		
**Lives with tobacco user** (Ref. No)	3129 (37.7)	2456 (37.8)	672 (37.3)	0.23	0.001
**Access to tobacco** (Ref. No)	1891 (22.8)	1632 (25.1)	243 (13.5)	107.88***	0.11
**Tobacco not allowed inside home** (Ref. No)	7086 (85.4)	5558 (85.6)	1527 (84.8)	0.14	0.004
**Discussed not using tobacco with parents** (Ref. No)	4087 (49.7)	3230 (50.2)	854 (47.7)	0.99	0.01
***Psychological factors***					
**Would enjoy using tobacco**, mean score ± SD	1.35 ± 0.64	1.36 ± 0.66	1.28 ± 0.53	5.08***	0.13
**Would enjoy using tobacco** (score)					
Strongly agree (1)	106 (1.3)	96 (1.5)	9 (0.5)		
Agree (2)	422 (5.1)	374 (5.8)	43 (2.4)		
Disagree (3)	1695 (20.5)	1307 (20.2)	390 (21.8)		
Strongly disagree (4)	6029 (73.1)	4683 (72.5)	1350 (75.3)		
**Would help reduce stress,** mean score ± SD	1.58 ± 0.79	1.60 ± 0.81	1.50 ± 0.73	5.24***	0.14
**Would help reduce stress** (score)					
Strongly agree (1)	202 (2.5)	173 (2.7)	28 (1.6)		
Agree (2)	962 (11.7)	796 (12.4)	162 (9.1)		
Disagree (3)	2256 (27.5)	1770 (27.5)	486 (27.3)		
Strongly disagree (4)	4788 (58.3)	3692 (57.4)	1102 (62.0)		
**Would help calm anger,** mean score ± SD	1.59 ± 0.81	1.61 ± 0.82	1.51 ± 0.75	4.67***	0.12
**Would help calm anger** (score)					
Strongly agree (1)	233 (2.8)	197 (3.1)	35 (1.9)		
Agree (2)	981 (12.0)	804 (12.5)	174 (9.8)		
Disagree (3)	2170 (26.5)	1718 (26.8)	450 (25.3)		
Strongly disagree (4)	4812 (58.7)	3698 (57.6)	1122 (63.0)		
**Would help enjoy parties,** mean score ± SD	1.44 ± 0.66	1.45 ± 0.67	1.38 ± 0.61	3.78***	0.1
**Would help enjoy parties** (score)					
Strongly agree (1)	91 (1.1)	76 (1.2)	14 (0.8)		
Agree (2)	516 (6.3)	431 (6.7)	82 (4.6)		
Disagree (3)	2290 (27.8)	1815 (28.1)	473 (26.5)		
Strongly disagree (4)	5338 (64.8)	4128 (64.0)	1216 (68.0)		
**ADHD** (Ref. No)	1300 (15.8)	1064 (16.5)	232 (13.0)	9.60**	0.03
***Behavioral factors***					
**Used alcohol** (Ref. No)	3302 (39.8)	2782 (42.8)	499 (27.8)	131.57***	0.13
**Used other substances** (Ref. No)	1598 (19.3)	1195 (18.4)	409 (22.7)	12.27***	0.04
**Initiated physical fights** (Ref. No)	770 (9.3)	548 (8.5)	229 (12.8)	60.05***	0.09
**Discussed tobacco on social media** (Ref. No)	1649 (24.6)	1273 (24.0)	380 (27.0)	2.52	0.02
***Primary outcomes***					
**Ever use** (Ref. No)	1795 (22.5)	1465 (23.4)	323 (18.8)	26.26***	0.06
**Current use** (Ref. No)	769 (9.7)	638 (10.3)	128 (7.5)	16.31***	0.05

Weighted frequencies (n), percentages (%), and means ± SD, are presented for the full sample and by race. ADHD: attention deficit hyperactivity disorder. Geographical regions were determined by the US Census. Pairs with the same alphabetical letter superscript are statistically different from each other.

* p<0.05;

** p<0.01;

*** p<0.001.

### Racial differences

Parental education was lower among Black than White participants, with significant racial differences found within each education level category (χ^2^=258.79, p<0.001). Significant regional differences were found as well (χ^2^=500.03, p<0.001). Black participants were more likely than White participants to be from the South (58.0% vs 35.1%), while the reverse was true for all other regions. Black participants thought it was easier to purchase tobacco (t=2.43, p<0.05) but were less likely to have access to tobacco in their parents’ homes (χ^2^=107.88, p<0.001; 13.5% vs 25.1%). Black participants were less likely than White participants to think that tobacco use had positive psychological benefits and were less likely to have been diagnosed with ADHD (χ^2^=9.60, p<0.01; 13.0% vs 16.0%). Black participants were less likely than White participants to have used alcohol (χ^2^=131.57, p<0.001; 27.8% vs 42.8%) but more likely to have used other substances (χ^2^=12.27, p<0.001; 22.7% vs 18.4%) and to have initiated physical fights (χ^2^=60.05, p<0.001; 27.4% vs 19.0%). Finally, Black participants were less likely than White participants to have ever used (χ^2^=26.26, p<0.001; 18.8% vs 23.4%) or to currently use a tobacco product (χ^2^=16.31, p<0.001; 7.5% vs 10.3%).

### Logistic regressions

All variance inflation factors were <2, ruling out multicollinearity as a source of bias.

*Ever use of tobacco products*

Model 1 in Table 2 displays the odds ratios (ORs), 95% confidence intervals (CIs), and p-values for the associations of each factor with ever use of tobacco for both Black and White participants. Factors that were significantly associated with ever use of tobacco for both Black and White participants include older age, male sex, living with an individual who uses tobacco, having access to tobacco in parents’ homes, thinking tobacco use would be enjoyable, thinking that tobacco use would help calm anger, having used alcohol, having used other substances, having initiated physical fights, and having an ADHD diagnosis. Geographical region was a significant factor for both Black and White participants, but not in the same direction. Among Black participants, those who resided in the Midwest and South were less likely to have ever used tobacco than those in the Northeast. Among White participants, those who resided in the Midwest, South, and West were more likely to have ever used tobacco than those in the Northeast. Peer influence (i.e. would use tobacco if asked by best friend) was only significantly associated with ever use among Black participants. Parental education, thinking that tobacco use would help reduce stress, and not discussing tobacco use on social media, were only significantly associated with ever use among White participants.

**Table 2:** Logistic regressions predicting ever use and current use of any tobacco product stratified by race

*Variables*	*Model 1: Ever use*						*Model 2: Current use*					
	*Non-Hispanic White (N=4952)*			*Non-Hispanic Black (N=1302)*			*Non-Hispanic White (N=4905)*			*Non-Hispanic Black (N=1288)*		
	*OR*	*95% CI*	*Rank*	*OR*	*95% CI*	*Rank*	*OR*	*95% CI*	*Rank*	*OR*	*95% CI*	*Rank*
**Sociocultural factors**												
Age (years) 12–14 (Ref. 15–17)	0.4***	0.4–0.5	6	0.5*	0.3– 0.99	5	0.3***	0.2–0.4	4	0.4	0.2–1.1	-
Male (Ref. Female)	1.2**	1.1–1.4	14	2.1**	1.3–3.4	9	1.0	0.9–1.1	-	1.9	0.9–4.2	-
Parental education level	0.8***	0.7–0.9	9	1.0	0.9–1.1	-	0.9**	0.8–0.9	7	1.0	0.9–1.2	-
Midwest (Ref. Northeast)	1.5***	1.4–1.5	15	0.6***	0.5–0.6	13	1.1***	1.1–1.2	11	0.6***	0.5–0.8	6
South (Ref. Northeast)	1.8***	1.8–1.9	13	0.6***	0.6–0.7	10	0.9***	0.9–1.0	10	0.6***	0.5–0.7	7
West (Ref. Northeast)	1.4***	1.3–1.4	16	1.0	0.9–1.0	-	0.8***	0.8–0.9	9	1.2	0.9–1.5	-
Would use if asked by best friend (Ref. No)	1.5	0.9–2.4	-	1.9*	1.1–3.2	6	1.4	0.9–2.3	-	1.2	0.6–2.6	-
Difficulty purchasing tobacco	0.9	0.8–1.0	-	0.9	0.8–1.0	-	1.0	0.8–1.2	-	1.0	0.8–1.1	-
**Household environment factors**												
Lives with tobacco user (Ref. No)	1.7***	1.4–2.1	7	1.6**	1.1–2.2	8	1.6***	1.3–1.9	6	1.3	0.8–2.2	-
Access to tobacco (Ref. No access)	1.5***	1.3–1.8	8	1.2*	1.0–1.5	11	1.1	0.9–1.5	-	2.0***	1.4–3.0	5
Tobacco not allowed inside home (Ref. Tobacco allowed)	0.9	0.8– 1.1	-	0.5	0.2– 1.2	-	0.9	0.7–1.2	-	0.6	0.3–1.5	-
Discussed not using tobacco with parents (Ref. Not discussed)	1.0	0.9–1.2	-	1.0	0.8–1.5	-	1.4**	1.1–1.7	8	1.0	0.9–1.1	-
**Psychological factors**												
Would enjoy using tobacco	2.5***	2.2–2.8	2	1.4***	1.2–1.8	4	3.8***	2.8–5.3	1	2.2***	1.8–2.8	2
Would help reduce stress	1.2***	1.1–1.3	5	1.2	0.9–1.5	-	1.0	0.8–1.3	-	1.3**	1.1–1.6	4
Would help calm anger	1.4***	1.3–1.5	4	1.3*	1.0–1.6	3	2.0***	1.8–2.2	2	1.6*	1.0–2.5	3
Would help enjoy parties	1.1	0.9–1.2	-	0.9	0.8–1.1	-	1.0	0.8–1.3	-	0.9	0.7–1.1	-
ADHD (Ref. No ADHD)	1.3*	1.0–1.7	12	1.6**	1.2–2.1	12	1.0	0.8–1.2	-	1.6	0.8–3.0	-
**Behavioral factors**												
Used alcohol (Ref. No)	2.8***	2.3–3.4	3	2.8***	2.1–3.7	2	1.2	0.8–1.9	-	1.7	0.8–3.8	-
Used other substances (Ref. No)	5.2***	4.0–6.7	1	4.2***	3.3–5.3	1	4.1***	2.8–5.9	3	7.3***	4.4–12.3	1
Initiated physical fights (Ref. No)	1.6**	1.1–2.2	10	1.9***	1.4–2.6	7	1.8**	1.2–2.7	5	1.3	0.6–3.0	-
Discussed tobacco on social media (Ref. No)	0.9*	0.8–1.0	11	1.1	0.8–1.4	-	1.0	0.7–1.3	-	0.8	0.3–2.0	-

Regressions were stratified by race. Sample size differences for each race are due to missing values. Rank: Order of importance of significant factors according dominance analyses.

*Current tobacco use*

Model 2 in Table 2 displays the odds ratios, confidence intervals, and p-values for the associations of each factor with current use of tobacco for both Black and White participants. Factors that were significantly associated with current tobacco use for both Black and White participants include geographical region, thinking tobacco use would be enjoyable, having used other substances, and having initiated physical fights. Differences by geographical region, however, were complex. Living in the South was significantly associated with a reduction in the odds of tobacco use for Black and White participants. Among Black participants, those living in the Midwest were less likely to currently use tobacco. Among White participants, those living in the Midwest were more likely to currently use tobacco. Living in the West was significantly related to lower odds of tobacco use for White participants, but had no relation to current tobacco use among Black participants. The availability of tobacco products in parents’ homes and thinking that tobacco use would reduce stress were only significantly associated with current tobacco use among Black participants. Older age, lower parental education level, living with an individual who uses tobacco, not being provided parental guidance in the past year about tobacco use, and having initiated physical fights, were only significantly associated with current tobacco use among White participants.

### Dominance analysis

*Ever use of tobacco products*

Model 1 in Table 2 provides the rank order of importance of the significant factors for Black and White participants for ever tobacco use. The DA ranked 13 significant factors among Black participants and 16 factors among White participants for ever use of tobacco. For both Black and White participants, the top four factors were identical, with minor variation in level of importance. These factors were the use of other substances, alcohol use, thinking that tobacco use would help calm anger, and thinking tobacco use would be enjoyable. Among the remaining significant factors, there were also many commonalities and a few distinctions. Among Black participants, peer influences ranked sixth and the male sex ninth in relative importance. Among White participants, peer influences were not significant, and male sex was ranked thirteenth in relative importance.

*Current tobacco use*

Model 2 in Table 2 provides the rank order of importance of the significant factors for Black and White participants for current use of tobacco. The DA ranked 7 significant factors among Black participants and 11 factors among White participants for current use. For both Black and White participants, the top three factors were identified with some minor variation in the level of importance. These factors were using other substances, thinking that tobacco use would help calm anger, and thinking tobacco use would be enjoyable. Among the remaining significant factors, there were a few commonalities and important distinctions. Among Black participants, thinking that tobacco use would reduce stress and having access to tobacco in parents’ homes were the fourth and fifth most important factors, followed by region. Among White participants, older age, having initiated physical fights, living with an individual who uses tobacco, lower parental education level, and not having received parental guidance about tobacco use, were the fourth through eighth most important factors, followed by region.

## DISCUSSION

These findings identify factors associated with Black adolescent tobacco use that are not associated with White adolescent tobacco use as well as Black–White differences in the relative importance of factors significantly associated with tobacco use. One of the most striking distinctions is the direction of regional differences for tobacco use. Black adolescents in the Northeast are more likely to use tobacco products compared to the South and the Midwest, while White adolescents in the Northeast are less likely to use tobacco products. In addition, peer influences, increased access to tobacco in parents’ homes, and thinking that tobacco use can help reduce stress, were significantly associated with tobacco use among Black but not White adolescents. These factors, in addition to the factors Black and White adolescents, have in common, should be considered in the development of strategies to prevent adolescent tobacco use in Black communities and address downstream tobacco-related health disparities. These findings indicate significant Black–White differences in the factors associated with tobacco use, which should be considered in the development of prevention strategies.

Overall, more factors were significantly associated with tobacco use among White than Black adolescents. These findings might be associated with the general lack of Black adolescent participants in tobacco research^23^. Like all epidemiological studies, the PATH Study carefully developed items based on evidence in the literature. If Black adolescents were not adequately represented in the literature, then factors unique to their experience might not be represented. Research conducted within Black communities might generate items and factors not present in the data but highly applicable to Black adolescents. These findings support more in-depth systematic investigations of factors critical to Black adolescent tobacco use.

These findings add to the growing body of research that indicates that the role of parental education differs between Black and White adolescents. However, the reasons for the difference remain unclear^17-19^. Assari et al.^18^ speculate that Black families might experience diminished gains from the sociocultural benefits of higher education due to systemic racism and discrimination: ‘Diminished gain is a phenomenon wherein the health effects of certain socioeconomic resources, and psychological assets are systematically smaller for Black individuals than White counterparts. These patterns are robust, with similar findings across different resources, assets, outcomes, settings, cohorts, and age groups. However, the role of diminished gain as a main contributing mechanism to racial health disparities has been historically overlooked’^11^. Others speculate that Black adolescents of higher SES might face unique environmental challenges, including discrimination in school, that increase the risk for tobacco use^19^. Together, these findings suggest that the relationship between race, SES, and tobacco use is complex. In-depth systematic investigations of SES-related factors are needed to understand its effects on Black adolescent tobacco use.

The rigor of the PATH Study procedures suggests that these findings are generalizable to Black and White adolescent populations in the US; however, two important nuances should be noted. First, there are significant inequities in the diagnosis and treatment of ADHD. Black children are less likely to receive a diagnosis or treatment of ADHD than White children^41^. Individuals with ADHD smoke at prevalence rates 2 to 3 times higher than the general population^42^. The presence of an ADHD diagnosis was associated with a significant increase in the odds of ever use among Black and White adolescents, but not current use. If the inequities in diagnosis and treatment were resolved, we speculate that the associations between tobacco use and ADHD might differ for Black adolescents. Second, relations between living with someone who uses tobacco (about 38%) and parental reports of access to tobacco in parents’ homes (22%) should be explored to understand if parents might be underestimating their children’s ability to access tobacco products at home.

### Strengths and limitations

This study’s strengths include data collected by a rigorous, landmark epidemiological study that oversamples Black adolescents and whose primary focus on tobacco use is consistent with the aims of this study. A strength also includes using dominance analyses to determine the relative importance of significant factors, which further explicated Black–White differences. Limitations include the cross-sectional design, which precludes the ability to infer causality. Also, this study utilized wave one of the PATH Study, collected nearly a decade ago and whose participants are now young adults. These findings provide a helpful juxtaposition with important findings using data from other sources collected during that time^43^. Still, the extent to which the findings apply to present day adolescents is unknown. Data from wave one and all new waves are released as they become available. Future research should build on these findings and examine longitudinal and intersectional racial and ethnic differences in factors associated with tobacco use, explore the viability of the putative intervention targets identified here, extend research on within-group differences, and examine measurement equivalence for all measures and items within Black communities.

## CONCLUSIONS

There are significant Black–White differences in the factors associated with tobacco use. Factors uniquely associated with Black adolescent tobacco use should be considered in developing strategies to prevent adolescent tobacco use in Black communities and prevent downstream health disparities.

## Data Availability

The data supporting this research are available from the following link: https://www.icpsr.umich.edu/web/NAHDAP/studies/36231
